# Selective Targeting of 4SO_4_-*N*-Acetyl-Galactosamine Functionalized *Mycobacterium tuberculosis* Protein Loaded Chitosan Nanoparticle to Macrophages: Correlation With Activation of Immune System

**DOI:** 10.3389/fmicb.2018.02469

**Published:** 2018-11-20

**Authors:** Nida Mubin, Mohd. Saad Umar, Swaleha Zubair, Mohammad Owais

**Affiliations:** ^1^Interdisciplinary Biotechnology Unit, Aligarh Muslim University, Aligarh, India; ^2^Department of Computer Science, Aligarh Muslim University, Aligarh, India

**Keywords:** Acr-1 (Rv2031c), *M. smegmatis*, RAW264.7, 4-SO_4_-GalNAc, CNPs

## Abstract

In the present study, we investigated potential of chitosan-based nanoparticles (CNPs) to deliver loaded therapeutic molecules to pathogen harboring macrophages. We fabricated stable CNPs employing ionic cross-linking method and evaluated their potential to target RAW 264.7 cells. The physicochemical characterization of as-synthesized CNPs was determined using electron microscopy, infrared microscopy and zeta potential measurement. Next, cellular uptake and intracellular localization studies of CNPs were followed in living RAW264.7 cells using confocal microscopy. We found that both Acr-1 loaded (CNP-A) and 4-SO_4_-GalNAc ligand harboring (CNP-L) chitosan nanoparticle experience increased cellular uptake by *Mycobacterium smegmatis* infected RAW cells. Following cellular digestion in model macrophage cell line (RAW), CNPs provide an increased immune response. Further, 4-SO_4_-GalNAc bearing CNP-L exhibits high binding affinity as well as antibacterial efficacy toward *M. smegmatis*. The data of the present study suggest that CNP-based nanoparticle offer a promising delivery strategy to target infected macrophages for prevention and eradication of intracellular pathogens such as *M. smegmatis*.

## Introduction

Tuberculosis, an important infectious disease, is a leading cause of death across the world. Approximately one-third of the world is infected with *Mycobacterium tuberculosis*, resulting in more than eight million new cases and two million deaths annually worldwide (Van Soolingen, 2001). Overwhelming numbers of both tuberculosis and non-tuberculous mycobacteria (NTM) have emerged as prevalent clinical entities (Espy et al., 2006). The situation is further complicated by the development of multiple antibiotic resistances among mycobacteria associated infections. Beside emergence of untreatable clinical isolates, failure in the prevention and eradication of mycobacterial biofilm poses a number of serious health issues (Yang et al., 2003). Biofilm formation has been elucidated as an important factor having significant presumption in pathogen virulence. Biofilm shields the pathogen from inhibitory effect of both antibiotics as well as immune cells (Hall-Stoodley and Stoodley, 2005). In order to develop effective strategies to curb this important disease, it is desirable to have a better understanding of mycobacterial pathogenesis, involved virulence factors and its interaction with host cell macrophages.

*Mycobacterium smegmatis*, with its short generation time and requirement of low biosafety level, serves as an appropriate model to study pathogenesis of genus Mycobacterium in general. Further, *M. smegmatis* shares important virulence gene homology with *M. tuberculosis* therefore might be helpful to understand different host-microbe aspects of virulent Mycobacteria (Altaf et al., 2010). Being relatively long-lived, macrophages are used as a persistent reservoir of invading *Mycobacterium* spp. (De Chastellier, 2009). Besides offering a safe heaven against antibody onslaught infected macrophages contribute to maintain subtherapeutic concentrations of antibacterial medications through continuous efflux of the antibiotics (Adams et al., 2011). Recently, nanotechnology has emerged as a platform for designing nanoparticles not only as a therapeutic delivery system for controlling mycobacterial infection, however, can be used as adjuvant in development of vaccine or therapeutics carrier for boosting immune response against *M. tuberculosis* infection.

The potential advantage of using nano-formulations over conventional therapies include their capacity to encapsulate or conjugate a variety of drugs/antigens thus offering tunable and site-specific targeted release of the entrapped solute (Weissleder et al., 2005). In addition, nano-system-based delivery system overcome biological barriers improve stability, solubility, bioavailability, and eventually facilitate sustained release of therapeutic substances. A wide range of nanomaterials have been used to deliver *M. tuberculosis* specific therapeutic agent (Dube et al., 2013). Among various nanoparticle-based delivery systems, chitosan co-polymers have been widely used as effective delivery vehicles (Meerak et al., 2013).

Chitosan-based nanoparticles (CNPs) have been reported to kill target bacterial cells by disturbing their cell wall. The interaction of CNPs with target bacteria ensues in leakage of cytoplasmic contents, inactivation of respiratory enzymes, and protein responsible for RNA and DNA replication. CNPs act like a “molecular knife” and contrive damage to bacterial cell wall even at low concentration (Rabea et al., 2003). The hollow, porous chitosan shell also permits high loading of therapeutics molecule. Additionally, CNPs safeguard degradation of entrapped therapeutics agent by apposing polyvalent layers on the surface of core entrapped material (Amidi et al., 2010). The targeted delivery of the in-house developed CNPs was achieved by conjugation of 4-SO_4_-GalNAc on the surface. The ligand 4-SO_4_-GalNAc is recognized by the C-type lectins (pattern-recognition receptor), a highly expressed receptor on the surface of phagocytic macrophages (Drickamer, 1992).

In the present study, we investigated uptake of as-synthesized CNPs by macrophages that eventually evoked host immune response against *M. smegmatis*. We evaluated functionalized CNPs for multipurpose applications. The focus of the study was to establish:

(1) Role of CNPs for its possible antibacterial and anti-biofilm activity against *M. smegmatis*.
(2) Immunological potential of Acr-1 protein loaded chitosan nanoparticles (CNP-A) upon interaction with the host macrophages.
(3) Potential of 4-SO_4_-GalNAc ligand bearing CNPs for their ability to enhance the immunological response against *M. smegmatis*.

## Materials and Methods

### Reagent

Chitosan (70 deacetylation) and 4-SO_4_-*N*-acetyl galactosamine (4-SO_4_-GalNAc), Tween 80, tripolyphosphate (TPP) 3-(4,5-dimethylthiazol-2-yl)-2,5-diphenyl tetrazolium bromide (MTT), sodium TPP dialysis bag (cut off mol. wt. = 12 kDa), antibiotic solution (penicillin/streptomycin, 0.1%v/v) were purchased from Sigma–Aldrich (St. Louis, MO, United States). Tissue culture media Dulbecco’s modified Eagle’s medium (DMEM) and fetal calf serum (FCS) were purchased from Gibco and plastic-wares were purchased from BD Biosciences (United States). Acr-1 was cloned, expressed, and purified following method described elsewhere (Normington et al., 1989). Other reagents and chemicals used were of analytical grade and obtained commercially unless stated otherwise. The triple distilled water was used in all experiments by a three-stage Millipore Milli-Q plus 185 purification system (Bedford, MA, United States).

### Cell Line, Animals, and Bacterial Culture Growth Condition

*Mycobacterium smegmatis* was cultured in 7H9 broth medium (supplemented with glycerol and tween-80) by shaking for 3 days at 37°C. Murine macrophage cell line, RAW264.7, was cultured in DMEM (HIMEDIA, Mumbai, India) supplemented with 10% fetal bovine serum, 1% penicillin-streptomycin solution, 1% L-glutamine, and HEPES [4-(2-hydroxyethyl)-1-piperazineethanesulfonic acid], at 37°C in humidified air supplemented with 5% CO_2_.

### Synthesis and Characterization of Acr-1 Protein Loaded Chitosan Nanoparticles

High-molecular-weight chitosan was used in synthesis of CNPs. The nanoparticles were prepared following published method as standardized in our lab (Ko et al., 2002). Briefly, chitosan solution (1 mg/ml) was prepared in 1% acetic acid and the mixture was stirred at 37°C to obtain a homogeneous solution. Dissolved chitosan solution containing 1% (w/w) Tween 80 was mixed with *M. tb* Acr-1 protein. Thereafter, 2 ml of an aqueous TPP solution (1 mg/ml) was added drop wise to the chitosan–Acr-1 solution with stirring. The resulting CNP-A were separated from suspensions by centrifugation for 30 min at 10,000 ×*g* with successive washes with deionized plain water. The pellet was dried and re-suspended in 0.1% acetic acid solution in normal saline. Plain chitosan nanoparticles (CNPs) were prepared following the same method as described above omitting inclusion of Acr-1 protein.

### Surface Modification of As-Synthesized CNPs With Ligand 4-SO_4_-GalNAc

The Acr-1 loaded chitosan NPs was chemically modified with 4-SO_4_-GalNAc employing method as reported earlier with slight modifications (Alex et al., 2011). In brief, 4-SO_4_-GalNAc was dissolved in 0.1 M acetate buffer (pH 3.0) and added to as-synthesized Acr-1 loaded chitosan suspension (CNP-A). The mixture was agitated at ambient temperature (30 ± 2°C) for 48 h to accomplish the reaction. The as-formed particles (CNP-L) were dialyzed against triple distilled water in dialysis bag (cut off mol. wt. = 12 kDa) for 18 h. Finally, an aliquot of nanoparticle (CNP-L) suspension was centrifuged for 30 min at 10,000 ×*g* and 4°C. The pellet was re-suspended in 100 μl of normal saline. The average particle size, polydispersity index (PDI), and zeta potential of the CNPs, CNP-A, and CNP-L nanoparticles was determined employing a Zeta-sizer 3000 (Malvern Instruments Ltd., Malvern, United Kingdom) in 5 mM HEPES (pH-7.4). The formulation was dispersed in low concentration phosphate buffer (5mM PO_4_, pH-7.4) for determination of zeta potential. The particle size distribution of the NPs was reported as a polydispersity index.

### Fourier Transforms Infrared Spectroscopy

Fourier transforms infrared spectroscopy (FTIR) was used to characterize as-synthesized CNPs, employing method published elsewhere (Kong and Yu, 2007). The chemical conjugation of 4-SO_4_-GalNAc with chitosan was confirmed by FTIR spectrum, recorded on FTIR multi-scope spectrophotometer equipped with spectrum v3.02 software.

### High-Resolution Transmission Electron Microscopy (HR-TEM)

The as-synthesized CNPs were subjected to morphological characterization; employing high-resolution transmission electron microscopy (HR-TEM; Hashimoto et al., 2004). A drop of various formulations of CNPs was placed onto a carbon-coated copper grid, forming a thin liquid film. For TEM analysis, a drop of aqueous suspension containing the CNP, CNP-A, and CNP-L-based NPs was put on carbon-coated copper grids to get a thin liquid film. The samples were dried and desiccated before loading onto a specimen holder.

### Percent Entrapment Efficiency (%EE)

For determination of actual amount of Acr-1 protein in formed CNPs, an aliquot of CNP-A (1 ml) was suspended in mixture of dimethyl sulfoxide (DMSO) and 0.1% acetic acid (1:20) and vortexed for half an hour followed by overnight magnetic agitation in the dark at 37°C; centrifuged at 10,000 ×*g* for 15 min. Complete solubilization of NPs caused release of entrapped Acr-1 in the surrounding solution. At various time intervals, the supernatant was decanted and released Acr-1 was determined employing micro BCA protein assay method (Agnihotri et al., 2004). A sample consisting of plain CNPs re-suspended in PBS was used as blank. The experimental set up was performed at least in triplicate. The amount of protein entrapped in the formed NP was determined following. Loading efficiency (LE) for Acr-1 was determined calculated as follows:

(1) $$\text{LE}=\frac{\text{Total amount of Acr-1}-\text{free Acr-1}}{\text{Total amount of Acr-1}}\times 100\%(w/w)$$

### *In vitro* Killing Assay to Determine Anti-mycobacterial Potential of As-Synthesized CNPs

To determine the anti-mycobacterial activity of various as-formed NPs, viz., CNP, CNP-A, and CNP-L, the NPs were co-incubated with *M. smegmatis* (4 × 10^5^ bacteria per well) in 7H9 broth medium in a 96-well plate. The optical density of the bacteria was adjusted to 1.0 at 600 nm. The total volume of the incubation mixture was kept 200 μl per well. Bacteria were harvested at the indicated time points and the number of colony-forming units (CFUs) present in aliquoted sample was determined. The experiment was performed in triplicate and values were averaged from three independent trials.

### Anti-mycobacterial Potential of As-Synthesized CNPs as Determined by Agar Diffusion Assay

*Mycobacterium smegmatis* culture was grown for 48 h in 7H9 medium and pelleted at 5000 ×*g* for 5 min. The final pellet was washed with 1 × PBS and re-suspended in 7H9 medium. The suspended culture (100 μL) was spread uniformly on 7H9 agar plates and incubated at 37°C for 30 min. Increasing amount of CNP, CNP-A, and CNP-L were loaded onto various wells along with control drug. The zone of inhibition was determined by measuring clear zone in bacterial lawn around corresponding well after 24–48 h of incubation (Bonev et al., 2008).

### Cytotoxicity Assay

RAW264.7 cells (1 × 10^5^ cells/ml) were grown in DMEM in a 96-well plate at 37°C in an atmosphere of 5% carbon dioxide (CO_2_) for 24 h. The cells were exposed to increasing concentration of various NP for 24 h. To determine the cell viability after 24 h exposure to various formulation, viz., CNP, CNP-A, and CNP-L, 20 μL of MTT stock solution (5.0 mg/ml in PBS, pH 7.4) was dispensed in each well. The plate was incubated for 4 h at 37°C in an atmosphere of 5% CO_2_ in the dark. The supernatant was aspirated carefully, and blue formazan crystals formed by the reduction of MTT were dissolved in buffer (sodium dodecyl sulfate [SDS] [11 g] in 0.02 M hydrochloric acid [HCl] [50 ml] and isopropanol [50 ml]). The absorbance was measured at 570 nm on a micro plate reader (Bio-Rad Model 550, United States). The experiment was performed in triplicate and mean value was used as the final representative result. Since absorbance is proportional to the number of living cells, cell viability was represented by the absorbance ratio of exposed cells to untreated control cells, which was expressed as a percentage. Chitosan-untreated cells in media were used as control (Fisichella et al., 2009).

### FITC Labeling of Nanoparticle and *M. smegmatis* Cell

Various as-synthesized chitosan NPs were labeled with fluorescein isothiocyanate (FITC) following procedure as published elsewhere (Lu et al., 2007). Briefly, CNP-based formulation (500 mg) was incubated with FITC (5 mg, dissolved at 2.5 mg/ml in DMSO) in 50 ml sodium carbonate buffer (0.1 M, pH 9.2) overnight at 37°C in the dark. Unreacted FITC was quenched by treatment with Tris buffer (10 ml, 1.0 M, pH 8.3) for 30 min. The labeled nanoparticles were extensively washed with sterile water until no color; residue remained followed by dehydration with absolute ethanol and acetone, and finally dried under vacuum in the dark at room temperature.

*Mycobacterium smegmatis* was labeled with TRITC following published protocol (Chassaing et al., 2011). Briefly, bacterial cells were suspended in 1 × PBS. TRITC (2.5 mg/ml in DMSO) was added to the bacterial cell suspension and stirred at room temperature in the dark for 3–4 h. Subsequently, Tris buffer (2.0 ml, 1.0 M, pH 8.3) was added, and reaction mixture was stirred for additional 15 min at room temperature to quench the free form of fluorescent labeling reagent. The labeled bacteria were centrifuged at 7000 ×*g* and dialyzed against water followed by lyophilization in dark.

### Cellular Uptake Study of As-Formed CNPs by RAW264.7 Cells Employing Confocal Laser Microscopy

The interaction of as-synthesized CNPs with RAW264.7 cells was followed by cellular uptake study employing confocal laser microscopy. Briefly RAW cells, seeded at a density of 5 × 10^5^ cells/cover slip using DMEM along with 10% FBS, were cultured at 37°C in a humidified atmosphere with 5% CO_2_. Various FITC-labeled CNPs, denoted as FITC-CNP, FITC-CNP-A, and FITC-CNP-L, were incubated with macrophage cells at 37°C. Uptake kinetics of as-synthesized nanoparticle by RAW264.7 cells was assessed in time-dependent manner (Wang et al., 2012). The cells were rinsed three times with 1 × PBS and fixed with 4% paraformaldehyde. For the nucleus staining, fixed cells were incubated with 1.0 mg/ml DAPI solution at 37°C for 30 min, prior to analyzing confocal microscopy. All the samples were examined under confocal laser scanning microscopy (LSM510 DUO, Zeiss).

### Antibacterial Potential of As-Synthesized CNPs Against Intracellular *M. smegmatis*

To examine antibacterial potential of as-synthesized CNPs against intracellular *M. smegmatis*, infected macrophages (5 × 10^5^ RAW cells) were treated with as-synthesized CNPs for 24 h. The RAW cells were exposed to *M. smegmatis* infection for 3 h prior to treatment. Control setups consisting of uninfected RAW264.7 were also exposed to as-synthesized CNPs. Extracellular bacteria were killed by the addition of gentamicin (20 μg/ml). After stipulated incubation, cells were washed and lysed with 0.5% Triton X-100; intracellular survival was estimated by plating serially diluted cultures on 7H9 agar plates and the colonies were enumerated after 72 h.

### Potential of As-Synthesized CNPs to Generate Intracellular ROS

Chitosan-based nanoparticles mediated generation of intracellular ROS in treated bacterial cells was estimated using 2’,7’ dichlorofluorescein diacetate (DCFDA) dye (Eruslanov and Kusmartsev, 2010). *M. smegmatis* cells (1 × 10^5^ cells/well) were seeded on coverslips. After 24 h, the bacterial cells were exposed to CNPs for 6 h. The un-interacted CNPs were washed off using 1 × PBS. Thereafter, the cultured cells were exposed to DCFDA dye (30 μM/ml) for 30 min at 37°C. The excess of DCFDA was removed following subsequent washing. The CNP treated experimental and control cells were visualized for qualitative analysis of ROS generation, employing fluorescence microscope (Zeiss model, United States).

### Anti-biofilm Potential of As-Synthesized CNPs

*Mycobacterium smegmatis* biofilm was cultured in polystyrene 96-well microtiter plates (Corning Inc., United States) following published procedure (Stepanoviæ, 2000). Briefly, overnight cultured *M. smegmatis* cells were suspended at the 1.0 absorbance (O.D._600_) in RPMI-1640 medium. After 3 days preformed biofilm was treated with 100 μg of the nanoparticle suspension/50 μL/well using 96-well microtiter plates. The plates were covered with lids and incubated at 37°C. After 36 h of treatment with CNPs, the plate was washed with PBS, and the biofilm activity was assessed by XTT [2,3-Bis-(2-methoxy-4-nitro-5-sulfophenyl)-2H-tetrazolium-5-carboxanilide] reduction assays. Biofilms were also observed by scanning electron microscopy (SEM) and confocal microscopy. The biofilm-harboring wells were washed with PBS buffer, fixed with glutaraldehyde, dehydrated with ethanol, and dried in vacuum desiccators. The samples were coated with gold and observed by a scanning electron microscope. The *M. smegmatis* biofilms for confocal microscopic studies were prepared employing similar methods, except that medium 7H9 also was used for culture.

### Live/Dead Staining of CNPs Treated *M. smegmatis* Biofilm

To study the effect of CNPs on biofilm inhibition, *M. smegmatis* was grown to an OD_600_ of 1.0 and diluted 1:100 in 7H9 broth medium. Aliquots of 600 μl (10^7^ CFU/ml) of diluted culture were transferred to a sterile 13 mm coverslip. The cells were exposed to MIC and sub-MIC concentration of CNPs. Control samples were prepared on a sterile glass coverslip with *M. smegmatis* cells only (positive) and 7H9 media only (negative) without any CNPs. The wells were washed twice with 3 m of sterile 0.9% saline. The cells were stained with a 1.5 ml mixture of 0.8 μM SYTO-9 green fluorescent dye and 10 μM propidium iodide (PI) red fluorescent dye of Live/Dead staining kit (LIVE/DEAD *Bac*Light Bacterial Viability Kit, Thermo Fisher Scientific, Waltham, MA, United States; Boulos et al., 1999).

### Effect of CNPs on *M. smegmatis* Biofilm as Revealed by Confocal Microscopy

Confocal microscopy was employed to assess effect of as-synthesized CNPs on *M. smegmatis* biofilm. The biofilm was grown on a cover slip. After 72 h, the preformed biofilms were treated with a sub inhibitory concentration of various CNPs. The treated biofilm was visualized by confocal microscopy using layer-scanning model. The treated *M. smegmatis* biofilm was washed with PBS, fixed with 4% formaldehyde, and stained for SYTO-9/PI staining for 1 h followed by washing step to remove unbound biofilm and dye, observed with a confocal microscope (LSM710, Zeiss, Germany).

### Effect of As-Synthesized CNPs on Expression of Co-stimulatory Molecules in Treated Macrophages

To study the role of various as-synthesized CNPs on expression of co-stimulatory molecule, 5 × 10^5^ macrophages cells in DMEM (supplemented with 10% FCS, and 2% HEPES) were seeded in each well of a 24-well plate. Bacteria were grown to an OD_600_ ranging from 0.6 to 0.8, passed through a 26-gauge needle three times and allowed to settle for 10 min. The infection was carried out at a multiplicity of infection (MOI) of 10:1 for 3 h in duplicate wells. The extracellular bacteria were removed by three washes using PBS. The cells were incubated in DMEM medium supplemented with 100 μg/ml gentamycin for 1 h and treated with various CNPs formulation. After 24 h of nanoparticle treatment at 37°C and 5% CO_2_, cells were recovered and stained with phycoerythrin-labeled fluorescent antibodies obtained from BD Pharmingen. Cell surface expression of CD80, CD86, CD40, and major histocompatibility complex (MHC) class II was assessed by flow cytometer (EPICS XL; Beckman Coulter; Dong et al., 1999).

### Statistical Analysis

Statistically significance of data among groups was determined using the Student’s *t*-test (two-tailed, equal variances).

## Results

### Characterization of Surface Decorated CNPs Employing FTIR and TEM

**FIGURE 1:**
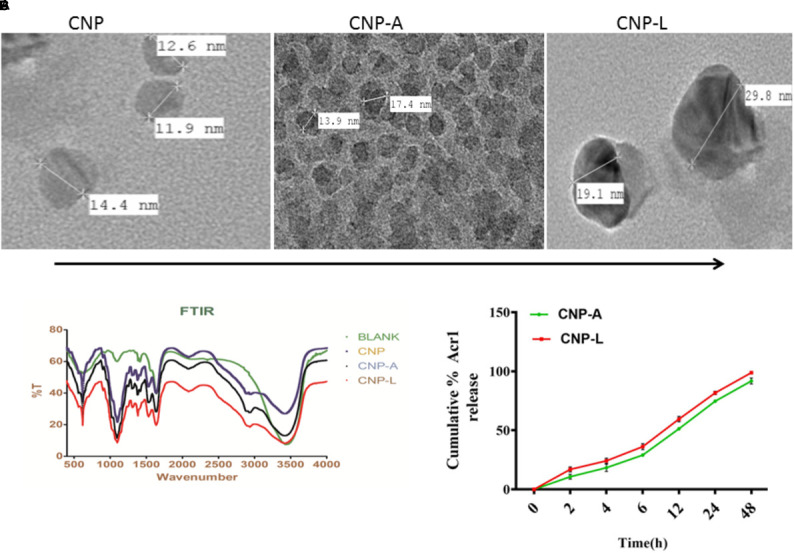
**(A)** Physical characterization of as-synthesized CNPs. Transmission electron microscopic images of CNP, CNP-A, and CNP-L (CNPs) nanoparticles (scale bar; 100 nm). **(B)** Fourier transformation infrared microscopy of CNPs (polydispersion index and zeta potential of CNPs as determined by Malvern Zeta Sizer). **(C)** Acr-1 antigen release from CNP-A/CNP-L nanoparticle at stipulated pH-5.5.

We employed chitosan and TPP as the “fixing materials” to fabricate chitosan shells through anionic and cationic electrostatic interactions. Tween 80 was used to prevent particle aggregation. TPP was used as the cross-linkers to form the CNPs particles. The resultant CNP-A did not allow leakage of entrapped Acr-1 protein. The TEM analysis suggests that the mean particle size of the CNPs in 5 mM saline was 17 and 29 nm for CNP-A and CNP-L nanoparticles as compared with 14 nm for unloaded CNPs (Figure 1A). The polydispersity index was 105 for plain CNP, 186 for CNP-A, and 226 for CNP-L, respectively. The PDI data indicated a narrow size distribution and a good colloidal stability of the as-synthesized NPs. The as-synthesized CNPs acquired positive surface charge. A significant increase in zeta potential was observed, when CNPs were conjugated with Acr-1 and ligand. CNP-A and CNP-L had a zeta potential of ±11.2 and 12.2 mV in 5 mM saline compared with ±9.01 for plain CNP. Next we assessed the zeta potential of macrophage co-cultured with CNPs. We observed highest zeta potential in CNP-L group (±30.2) as compared to both CNP-A (±26.3) and CNP (±24.5) group. The control macrophages (untreated) had a negative zeta potential value (-13.3 mV; Supplementary Figure S1).

### FTIR Spectroscopy

The FTIR spectroscopic studies of various as-synthesized CNPs have been shown in Figure 1B. The FTIR spectra of prepared chitosan and its conjugated form consist of various important absorption bands characteristics of various functional groups. The spectrum was recorded in the middle infrared wave number in 4000 to 500-1 cm^-1^ range. The amine NH- stretching vibrations were observed at 3500–3300 cm^-1^ and that for C-H were observed at 2900 in various CNPs groups. The absorption peaks at 1600–1000 cm^-1^ can be correlated to the presence of the C = O stretching of the amide I band. Bending vibrations of the N–H (N-acetylated residues, amide II band), C–H bending, OH-1 bending, respectively, were also observed in all three CNPs group. An upshifted new peak appeared at high wave number in both CNP-A and CNP-L between 1690 and 1630 and can be attributed to the amide C = = 0 stretch of Acr protein. The strong sharp peak in 3400–2900 cm^-1^ was also observed in upshifted manner re-presenting amine NH stretch in both CNP-A and CNP-L. The observed broadening of peak at 3400 cm^-1^ in CNP-A and CNP-L can be correlated to hydrogen bonding. The spectrum showed the characteristic peaks of the substituted secondary amide in the 3300–3400 cm^-1^ region. The peaks observed at 1100 to 600 cm^-1^ can be attributed to the C–S stretching vibrations of sulfides and disulfides bonds in CNP-L (Figure 1B).

### Encapsulation Efficiency of CNP-A Nanoparticles

Earlier reports suggested that the encapsulation efficiency of antigen with nanoparticle was due to the electrostatic and hydrophobic interaction between the Acr-1 protein and CNP and this could be a reason for the increase in size of the particle (Gan and Wang, 2007). Figure 1 represents the TEM images of the CNP-A nanoparticle that showed some structural changes when loaded with Acr-1 (Figure 1A). The entrapment efficiency of the Acr-1 in CNP was calculated and it was found that 79% of Acr-1 antigen was loaded in CNP. On the other hand when Acr-1 loaded chitosan (CNP-A) was conjugated with ligand, increase in size was observed in CNP-L (Figure 1A)

### *In vitro* Antigen Release

The profile of Acr-1 antigen release, under simulated acidic (pH-5.5) environment, from CNP-A loaded and CNP-L nanoparticles are shown in Figure 1C. The amounts of the antigen released from the CNPs after 6 h of incubation at pH 5.5 exceed 35% of the loaded protein. The particle showed good physical stability in acidic condition and their size did not noticeably change. The present result showed that under simulated phagosomal condition (pH-5.5) 70% of the loaded antigen was released after 12–14 h from CNP-A and CNP-L nanoparticle.

### Antibacterial Activity of Various As-Synthesized CNPs

**FIGURE 2:**
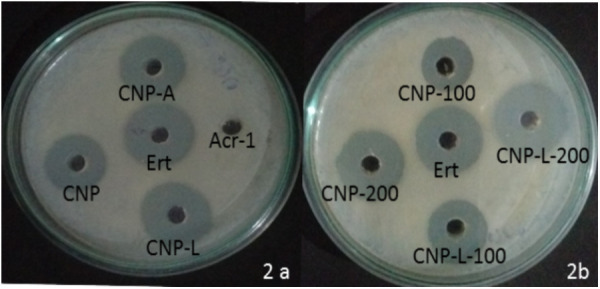
Dose-dependent killing of *M. smegmatis* by various as-synthesized CNPs. **(A)** Antibacterial activity of various CNPs-based formulation was assessed by agar diffusion method: various CNPs (CNP, CNP-A, and CNP-L) were loaded at 100 μg/20 μL dose into wells of the plate exposed to *M. smegmatis*. Growth inhibition was determined by measuring the zone of inhibition formed in bacterial lawn after 48 h; erythromycin was used as a control. **(B)** *M. smegmatis* culture was incubated with increasing concentrations of both CNP and CNP-L group to study dose-dependent killing efficiency of as-synthesized CNPs.

The antibacterial activity of various as-synthesized CNPs against *M. smegmatis* was assessed following published protocol (No et al., 2002). Exponentially grown bacteria were incubated with increasing concentrations of various CNPs. The MIC of various CNPs was determined using serial twofold dilution against bacterial inoculum of 1 × 10^8^ CFU/ml. The positive control consisted of 7H9 broth medium with tested bacterial inoculum and negative control contained only broth. The MIC is the minimal concentration of antimicrobial agent that visually inhibits 99% growth of bacteria. The MIC was assessed on the basis of visual turbidity of the tubes both before and after incubation of as-synthesized CNPs. The residual bacterial load was assessed by harvesting bacteria at various time points and plating serial dilutions of suspension on 7H9 agar plates. The residual live bacteria were enumerated after 72 h incubation. The MBC was observed to ensure presence or absence of bacterial growth in 7H9 agar plates. The MBC endpoint is defined as the lowest concentration of antimicrobial agent that kills 99.9% of the initial bacterial population (Supplementary Figure S2). CNP-A formulation showed less antibacterial activity as compared to plain CNP and CNP-L in significant manner (Figure 2A) The observation suggests that Acr-1 inhibits antibacterial activity. Both CNP and CNP-L nanoparticles treatment showed significant inhibitory activity against *M. smegmatis* in dose-dependent manner (Figure 2B). The anti-mycobactericidal activity of various as-synthesized CNPs was also assessed by Agar diffusion method. All synthesized CNPs were evaluated for their antibacterial activity against 10^6^ CFU/ml of the *M. smegmatis* on agar plate. After 72 h, all three NPs, viz., CNP, CNP-A, and CNP-L showed a clear zone of inhibition. The plate loaded CNP-A group demonstrated less zone of inhibition as compared to CNP and CNP-L formulation. Acr-1 protein alone failed to impart any antibacterial activity, as suggested by a dense population of bacteria in agar plate. Interestingly, CNP-A contribute important role in immuno-stimulation of the host by evoking immune response against *M. smegmatis* (Figure 8).

### CNPs Mediated Damage of *M. smegmatis* Cell Wall

**FIGURE 3:**
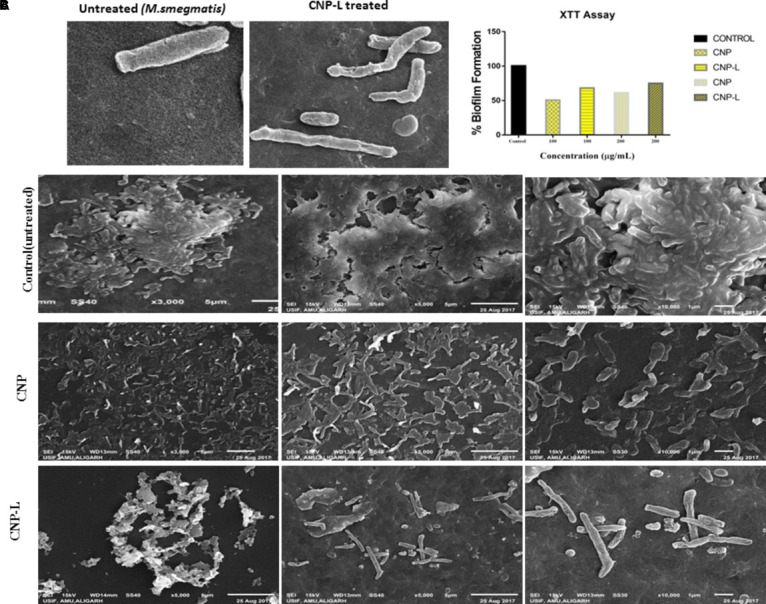
CNPs treatments disintegrate the *M. smegmatis* cell wall. **(A)** Scanning electron microscopic images showing disruption in cell wall morphology of single *M. smegmatis* (control) and after exposure to as-synthesized CNP-L (100 μg/ml) for 36 h. **(B) (i)** Control *M. smegmatis* biofilm (untreated), **(ii)** CNP treatment inhibits the biofilm formation, and **(iii)** CNP-L treatment at the same dose showed more biofilm inhibition after 36 h. **(C)** Biofilms were grown and treated with CNP and CNP-L followed by incubation with XTT for 1 h, absorbance at 495 nm was measured for biofilm inhibition. Increased killing of *M. smegmatis* was observed at 200 μg/ml dose of CNP-L. *Note*: scanning electron microscopy analysis was performed on a SU1510 scanning electron microscope (Hitachi, Tokyo, Japan).

Scanning electron microscopy was used to study interactions between CNPs and *M. smegmatis* (Figure 3A) shows that *M. smegmatis* cells lost it cellular integrity after exposure to CNP and CNP-L treatment at a concentration of 100 μg/ml whereas untreated cells (control group) remained intact (Je and Kim, 2006). Longer treatment with CNPs resulted in the formation of large sized bacterial aggregates. Next we assessed the potential of pre-formed CNP and CNP-L nanoparticles to inhibit pre formed *M. smegmatis* biofilm under *in vitro* conditions by monitoring SEM analysis. We observed that both CNP groups were efficient to inhibit biofilm mass after 36 h treatment. CNP-L treatment was more effective as compared to CNP at the same dose in terms of biofilm inhibition (Figure 3B).

### Intracellular ROS Production by As-Synthesized CNPs

**FIGURE 4:**
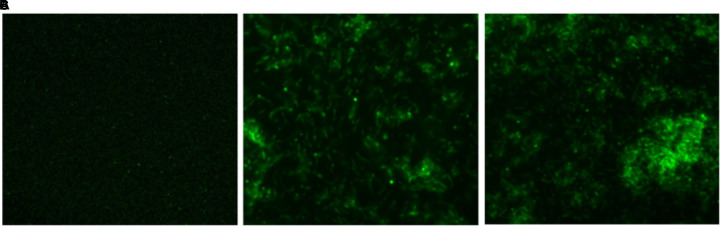
Fluorescence micrograph showing the generation of intracellular reactive oxygen species (ROS) using DCFDA dye in CNPs treated *M. smegmatis* cells **(A)**. Control **(B,C)** cells exposed to CNP and CNP-L at prefix MIC dose for 6 h showing increases in fluorescence intensity demonstrating excess generation of ROS production.

In order to determine intracellular ROS production by CNPs, we followed fluorescence probe DCFH-DA employing fluorescence microscope. DCFDA is cell permeant indicator and employed to probe ROS. The acetate groups of the DCFH-DA are removed by intracellular esterases to form a non-fluorescent product 2’,7’ dichlorofluorescein (DCFH). The DCFH dye reacts with generated reactive oxygen species (ROS) to produce the fluorescent product 2,7 dichlorofluorescein (DCF) which gets trapped within the cells making it fluorescent. A significant quantitative concentration-dependent increase in % ROS was observed increase in the form of fluorescence on treatment with as-synthesized CNPs at 100 μg/ml (Figure 4).

### Assessment of Biofilm Inhibition by As-Synthesized CNPs Using SYTO-9 Dye

**FIGURE 5:**
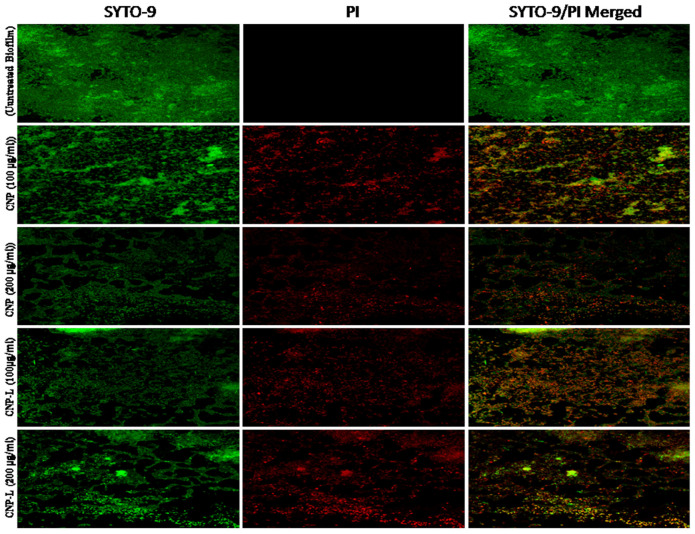
Confocal microscope image showing *M. smegmatis* biofilm inhibition by CNPs as visualized in 63X oil immersion magnification: anti-biofilm activity of CNPs was assessed by incubating *M. smegmatis* with increasing concentrations of CNPs for 36 h in a six-well plate. The treated biofilm was stained with SYTO-9/PI. The addition of increasing concentration (100–200 μg/ml) of CNPs inhibited *M. smegmatis* biofilm formation. Red-dye showing PI-stain corresponds to killing activity, and Green dye showing viable bacteria in pre formed biofilm. Yellow color corresponds to co-localization of merged green and red dye at same place.

Biofilms limit diffusion of drug molecules and proffer to the pathogenesis of underlying infection. The biofilm inhibition is crucial to halt bacterial colonization and also to increase the susceptibility of bacteria against given therapeutic agent. *M. smegmatis* is known for its ability to form biofilms (Zambrano and Kolter, 2005). We determined potential of CNPs to inhibits biofilm formation (and/or disrupted the preformed biofilms) under *in vitro* conditions by monitoring the binding of the SYTO-9 dye to live bacterial cells (SYTO-9 Stained cell – green color). As Acr-1 protein did not impart any antimicrobial activity, we avoid including CNP-A group in biofilm treatment studies. To assess the effect of CNP treatment on disruption of preformed biofilms, *M. smegmatis* biofilms were grown for 48 h in a six-well microtiter plate. The biofilm was exposed to increasing concentration of CNPs. Significant, disruption of preformed biofilms was observed in CNP and CNP-L nanoparticle treated group (killed bacteria were stained with PI-Red stain; Figure 5). In contrast, early exposure of *M. smegmatis* to CNP and CNP-L NPs resulted in significant inhibition of biofilm formation. Our confocal data showed that both CNP and CNP-L at dose of 100 μg/ml treatment for 36 h decreased more than 50 and 60% of the biofilms inhibition, respectively. Whereas exposure with both CNP and CNP-L at the dose 200 μg/ml, dose resulted in more than 60 and 65% killing of bacteria, respectively (Figure 3C).

### Safety Evaluation and Cytotoxicity Assay

**FIGURE 6:**
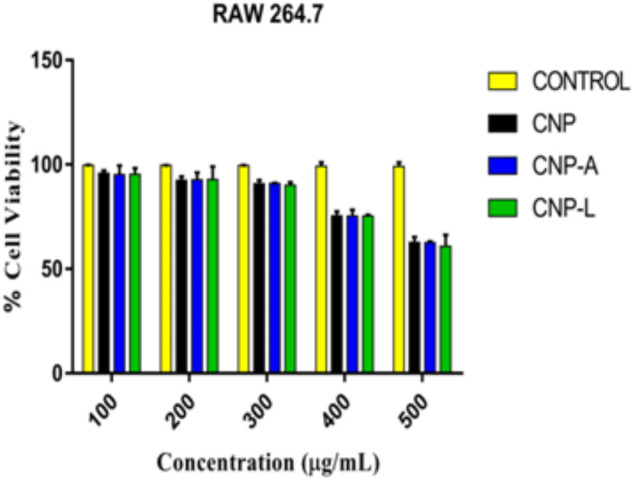
Assessment of cytotoxic activity of various as-synthesized CNPs against mouse macrophages 264.7 cells. Macrophages were treated with increasing concentration of CNPs for 24 h: cell viability was determined by MTT assay. Experiments were performed in triplicate; results are shown as mean ± SD.

Despite potent antibacterial activity of chitosan, the use of CNP as therapeutic agents is limited. It can be speculated that chitosan conjugation with ligand (4-SO_4_-GalNAc) coating resulted in modulation of host immune cell component. As *M. smegmatis* is an intracellular pathogen, we used the MTT assay to test the cytotoxicity of various CNPs in a dose-dependent manner on RAW264.7 cell line (Kuo et al., 2005). In MTT assay, metabolically active cells reduce MTT to purple formazan; the intensity of dye read at 570 nm is directly proportional to the number of viable cells. The cytotoxicity assay showed a dose-dependent drop in cell viability (Figure 6). The cell viability was not significantly affected at 24 h of incubation in the presence of 100–200 μg/ml a dose that was found to be lethal for the bacteria tested in this study. Cell viability dropped drastically at the dose of 500 μg/ml. We also examined the macrophages cell morphology in a monolayer culture after the treatment with varying concentrations of CNP, CNP-A, and CNP-L NPs. Microscopic observations showed no distinct morphological changes in the cells treated with a bactericidal dose (200 μg/ml) of CNPs group.

### Cellular Uptake of CNPs by RAW264.7 Cell

We followed interaction of CNPs with macrophages in context of surface charge (zeta potential) of the target cells. Our goal was to determine cellular binding of nanoparticle with macrophage membrane. All cells generate an electrical potential across their plasma membrane driven by concentration gradient of charged ions. NPs used in diagnostic and therapeutic applications are treated as inert probes or delivery vehicles for infectious diseases. While NP-delivered therapeutic agent expected to alter a cell, it is assumed that the NP itself will not change the biological system as such. Previous reports had shown that the cellular binding of NPs is affected by membrane potential (He et al., 2010). We first examined the membrane potential of RAW264.7 cells in order to study the effect of binding propensity of CNPs to the plasma membrane independent of internalization. Membrane potential of intact macrophages cell was checked followed by CNPs internalization. Cellular binding of CNPs with macrophages cell showed increased positive potential.

**FIGURE 7:**
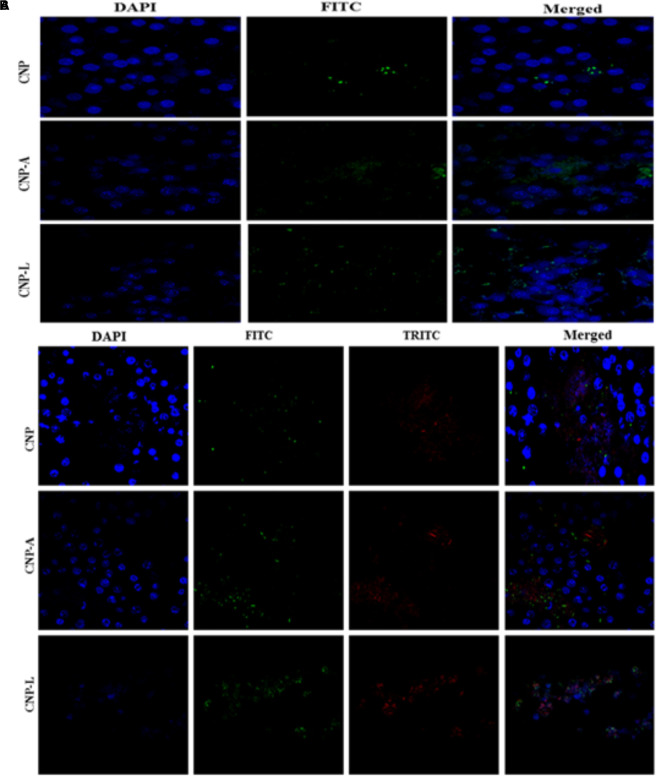
Confocal microscope image showing cellular uptake capacity of ligand conjugated chitosan nanoparticles by RAW264.7 mouse macrophages cell line: macrophage cells were treated with equal concentration of CNP, CNP-A, and CNP-L NPs for 4 h and checked for co-localization of NP in cells. **(A)** Low uptake of various as-synthesized various CNPs was observed in uninfected RAW macrophages **(B)** while *M. smegmatis* infected RAW cells were more susceptible to engulf CNP-L as compared to both CNP and CNP-A nanoparticle. Staining is as follows: blue–DAPI–stained nuclei, green–FITC labeled CNPs, and red–TRITC labeled *M. smegmatis* cells. Post 4 h incubation, more no of CNP-L was seen inside the cytoplasm as compared to CNP-A and CNP. Scale bar shows 10 μm. Pearson’s coefficients were calculated and expressed as mean value ± SD for at least three images obtained in three independent experiments.

Next, we employed confocal microscopy study to investigate the interaction of NPs with RAW cells. RAW264.7 cells were treated with varying concentration of FITC-labeled CNP, CNP-A, and CNP-L for 4 h (Figure 7A; He et al., 2010). Confocal microscopy images demonstrated that FITC-CNP-A and FITC-CNP-L NPs are efficiently taken up by infected macrophages as compared to plain CNP group. The uptake of CNP-A and CNP-L by RAW264.7 cells was at least five times more to that of plain CNPs. The data highlighted representative images of live cells incubated with FITC-CNP, CNP-A, and CNP-L for 4 h in RAW264.7 control cells and TRITC-tagged *M. smegmatis* infected macrophages cell. While in untreated control cultures (Figure 7A), low fluorescence was detected. Infected cells treated with FITC-CNP-L NPs, fluorescence intensity increased with the NPs uptake (Figure 7B). The lowest fluorescence intensity was observed in control CNP group tested, high level of cellular uptake was observed in CNP-L group, at the same dose. The results support the data and demonstrate that the FITC-CNP-L conjugated NPs were efficiently and rapidly taken up by the infected cells demonstrating the role of galactose residue ligand for specific recognition by C-type lectins, mannose receptor highly expressed on infected macrophages. In addition, the confocal analysis allowed us to visualize the intracellular distribution of the fluorescent Acr-1 (either encapsulated in chitosan alone and along with surface modified galactosylated CNP) into the macrophage cells. Figure 7B shows that the fluorescence is distributed inside the cells, confirming the internalization of the NP in infected cell. By comparing the cellular uptake of CNP, CNP-A, and CNP-L, the uptake of CNP-L (Figures 7A,B) in both uninfected control cells and infected cells, we again observed a higher rate of delivery of FITC–CNP-L NPs confirming the targeted delivery of therapeutic molecule through ligand targeted mannose receptor in infected macrophage cells.

### Efficacy of CNPs in Killing of Intracellular *M. smegmatis* and Also Upregulation in Expression of Co-stimulatory Molecules in Infected Macrophages After Treatment With CNPs

Macrophages are professional phagocytic cells that can internalize particles up to 10 μm in size. Keeping this fact into consideration, we hypothesized that exogenous addition of CNPs groups may lead to endocytosis of all modified nanoparticles by macrophages, resulting in intracellular killing of bacteria. Fluorescence microscopy was used to follow internalization of FITC-labeled CNPs by TRITC-*M. smegmatis* infected macrophages. CNP internalization was followed in both infected as well as uninfected macrophages under identical conditions. The control macrophages (uninfected) showed minimal FITC-CNPs internalization; however, the TRITC-*M. smegmatis* infected macrophages showed active endocytosis of fluorescently labeled CNP-L NPs as compared to CNP and CNP-A NPs group (Figure 7B).

**FIGURE 8:**
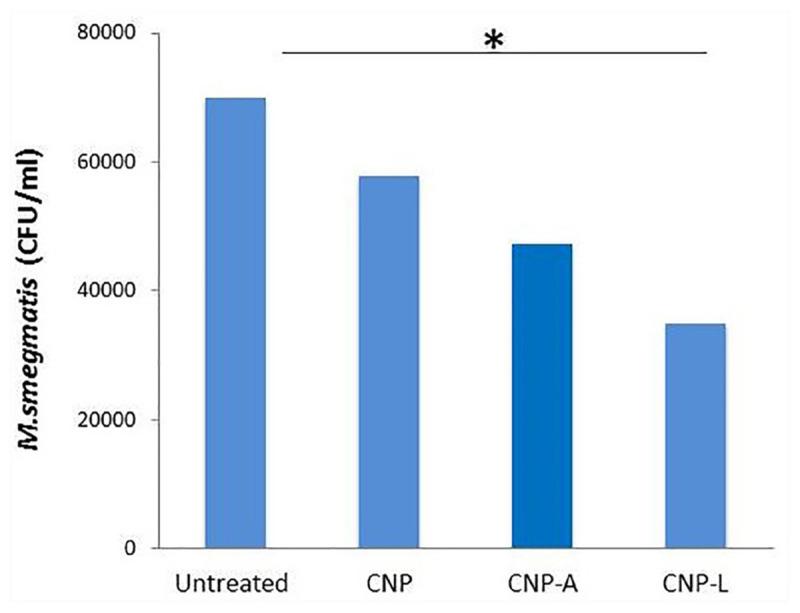
Chitosan-based conjugated nanoparticles (CNPs) kill intracellular *Mycobacterium smegmatis*. RAW264.7 macrophage cells were incubated with various CNPs formulation for 24 h after *M. smegmatis* infection. Macrophages infected with bacteria alone were used as a control. The cells were lyzed after 24 h post-infected macrophages and intracellular bacterial survival was determined by determining colony-forming unit (CFU) assay. Experiments were performed in triplicate; results are shown as ± SD; ^∗^*P* ≤ 0.05.

The infection conditions and intracellular survival kinetics of *M. smegmatis* had already been well characterized in macrophages (Anes et al., 2006). For this reason, we chose this model to evaluate the killing activity of *M. smegmatis*-infected macrophages after 24 h treatment with various CNPs. Macrophages were infected with *M. smegmatis* infection, before 3 h prior to interaction with various CNPs at prefixed dose. The concentration of nanoparticles used in the study was found to be non-toxic to the macrophages (Figure 6). A significant reduction in intracellular bacterial survival was observed after 24 h treatment with CNP-L and CNP-A in terms of CFU count after plating on 7H9 agar as compared with untreated macrophages (Figure 8). Treatment with CNPs had no effect on the phagocytosis of *M. smegmatis* by RAW cells.

**FIGURE 9:**
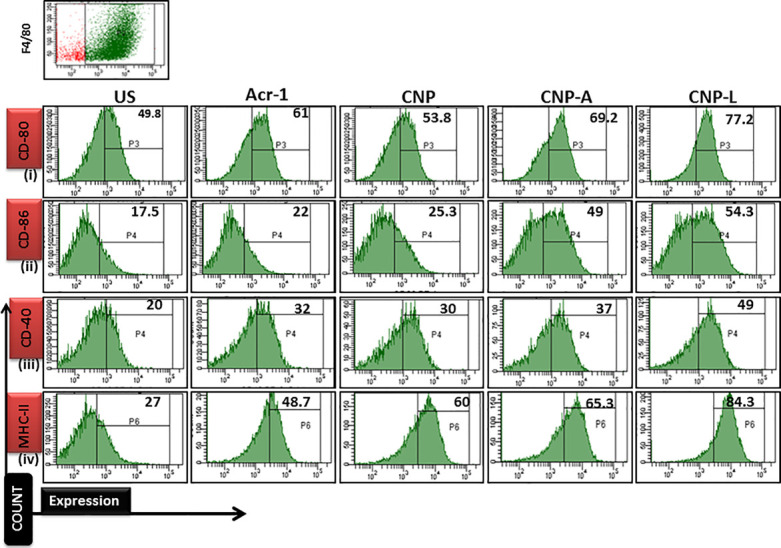
Flow cytometry analysis of co-stimulatory molecule in *M. smegmatis* infected macrophages cell line: post 24 h treatment with all CNP, CNP-A, and CNP-L groups, infected macrophages cell were acquired and checked for costimulatory molecule expression by FACS analysis. **(i–iv)** showed that CNP-L group are more efficient to enhance upregulated expression of co-stimulatory molecule CD-40/80/86/MHC-II, expressed on *M. smegmatis* infected macrophage cell as compared to CNP and CNP-A group.

Next, we checked how Acr-1-based nanoparticles can enhance immune response against *M. smegmatis* infection. The infected macrophages undergo subtle changes upon establishment of *M. smegmatis* infection. The optimum expression of both MHC as well as co-stimulatory molecules on APCs is imperative in deciding its future interaction with effector T cells in terms of their activation (Demotz et al., 1990). Many reports suggest adjuvant potential of CNPs against infectious diseases (Prego et al., 2010). The confocal data demonstrated that uninfected macrophages are less efficient in CNP intake while infected macrophages are more efficient in uptake of CNP-L. Further, the flow cytometer data showed that both CNP-A and CNP-L nanoparticles successfully upregulate expression of CD-80/86/40/MHC-II molecule on RAW264.7 cells (Figure 9).

## Discussion

Mycobacterium pathogens adapt intracellular mode of parasitism as a strategy to counteract antibody onslaught. Incidentally intracellular abode also help them to withstand antibiotics used as a modality in tuberculosis treatment, as they fail to access bacteria residing inside the cells (Wakamoto et al., 2013). The situation needs development of drug delivery system that can deliver effective antibiotic directly inside the infected cells (Wu et al., 2005). In recent times, nanomedicines have received a great deal of attention as a therapeutic agent because of their unique mode of action (Moghimi et al., 2005). The data of the present study suggest that a specific dose of CNPs can inhibit or kill the mycobacterium without harming the host cells. We prepared CNPs and assess their anti-mycobacterial activity. It was found that plain CNPs possess strong antibacterial activity, which depends on a multiple factors, viz., chitosan molecular weight; the viscosity, ionic strength, pH, and presence of metallic ions in the medium; the temperature; and the concentration of chitosan in composite preparations (Rinaudo et al., 1993).

Next, we supplemented CNPs with Acr-1 protein and evaluated the antibacterial as well as immunomodulatory role of the combination. We also surface decorated CNP-A with a ligand (4-SO_4_-GalNAc) that recognized C-type lectins, surface molecule of macrophages or other APC (McGreal et al., 2005). Various as-synthesized CNPs used in this study were found to be stable at both physiological as well as at 4°C up to at least for 1 month time period even when present in solution.

Under specific conditions, chitosan provided a hollow cage like structure that protects Acr-1 and also coerce a strong interaction with ligand on the periphery of nanocomposites (Li et al., 2008). Chitosan binds to metal ions of ligand because of the presence of amine and hydroxyl groups. The as-synthesized CNPs, as determined by FTIR-spectra and TEM studies, were found to be spherical and remained stable for a longer duration. (Figures 1A,B).

The present study showed distinct differences in the susceptibility of *M. smegmatis* strain to various as-synthesized CNPs. CNP and CNP-L were found to be more effective in killing of *M. smegmatis*. In contrast, CNP-A was not significantly effective in killing of *M. smegmatis* due to unequal proportion of chitosan vs Acr-1 protein ratio as compared to other groups hence proving Acr-1 antigen has not manifested toxicity issue. The interaction of as-synthesized CNPs and *M. smegmatis* could be correlated to cationic chitosan and the negatively charged molecules present on bacterial cell wall (Raafat et al., 2008). In contrast, the mycobacteria cell wall is rich in lipids such as mycolic acids and thus limits the binding and permeability of drug molecules (Nikaido, 2003). To study the mechanism by which CNPs inactivate bacterial cells components, the effect on cell integrity and disturbance was evaluated by SEM (Figure 2). The data indicated that irreversible damage could be induced on mycobacterial cells after direct contact with CNPs and CNP-L treatment. The damage to the cell may be caused by interactions of CNP and CNP-L with free amino group- and sulfur-containing compounds (Sahariah and Masson, 2017). Both core chitosan and surface ligand moiety tend to have a high affinity for such molecules. The inhibitory effect could also be due to diffusion of CNPs through the channels present in the biofilms (Wilking et al., 2013). Taken together, the data indicate that chitosan-based CNPs not only exhibit potent anti-mycobactericidal activity but also impede the biofilm formation.

Mycobacterium has been reported to synthesize exopolysaccharides that protect it from the host cell offensives. The anti-biofilm activity could be correlated to inhibition of exopolysaccharide synthesis it has been shown that CNPs impair exopolysaccharide synthesis in *M. smegmatis* cells limiting the formation of biofilm (Hall-Stoodley and Stoodley, 2005). The inhibition of biofilm can also be linked to inhibition in iron uptake responsible for biofilm formation in Mycobacteria. This distinction could be because of the presence of abundant LPS, which leads to strong interaction with CNP, produced by *M. smegmatis.*

The present study further demonstrated that CNPs treatment lead to generation of ROS production in *M. smegmatis*. The direct correlation between ROS generation and oxidative stress may lead to DNA damage, further suggest as an important route by which CNPs treatment causes DNA damage leading to inhibition or killing of *M. smegmatis* cells.

An important aspect for any molecule to be used as a therapeutic agent is that it should eliminate the target cells without affecting the viability of neighboring host cells. Many medically relevant polymeric nanoparticles such as chitosan biomaterial have been investigated for their cytotoxicity, but different researchers have reported varying effects for these nanoparticles on mammalian cells. The observed differences have been largely attributed to individual CNPs preparation methodologies and target cells.

The interaction of CNPs with mouse macrophages cell line RAW264.7 resulted in cell lysis at higher dose of chitosan treatment, while there was no harm to host macrophage cells up to dose of 100–300 μg/ml. Interestingly, the same dose was found to have cytotoxic effect to *M. smegmatis* after 72 h of treatment. The formulation CNP-L that harbored a modifier at the particle surface has been shown to have a significant inhibitory effect against *M. smegmatis* strain. In contrast, CNP-A was found to have very less antimicrobial effect against *M. smegmatis* strain.

Macrophages can readily internalize foreign material ranging up to 10 μm in size owing to their phagocytic function. Macrophages have been shown to be an ideal candidate for transporting antibacterial therapeutic molecule to various parts of the body. Since mycobacteria are an intracellular pathogen that halt the endo-lysosomal fusion and reside in the phagosomal compartments of macrophages, it is important to deliver the therapeutic molecules to the target sites that would otherwise be inaccessible due to the presence of physical barriers (Levitte et al., 2016).

We also observed active uptake of FITC-labeled CNPs by macrophages. CNPs uptake by the RAW264.7 cells was executed through C-type lectin receptors mediated endocytosis. The elevated intracellular killing of *M. smegmatis* could be because of the delivery of endocytosed CNP-L and CNP-A to macrophages phagosome, where *M. smegmatis* resides. Alternatively, it could be because of the prolonged exposure (24 h) of CNPs treatment and release of Acr-1 from the CNP-A (Koppolu and Zaharoff, 2013). Treatment with CNPs upregulates the expression of co-stimulatory molecule such as CD80/86/40/MHC-II. The upregulation of co-stimulatory molecule is necessary for optimal activation of immune response that eventually results in elimination of pathogen.

## Conclusion

The present study suggests that CNPs and CNP-L exhibit potent anti-mycobacterial activity against intracellularly residing *M. smegmatis*. CNP mediated mycobacterial cell inhibition may be attributed to both cell wall disruption as well as inactivation of thiol-containing proteins. Both CNPs and CNP-L exhibit potent anti-biofilm activity against *M. smegmatis.* The MTT assay and cell morphology analysis results showed that various CNPs groups exhibit no discernible cytotoxic effect on macrophages at the bactericidal concentration. In contrast, treatment with higher doses caused significant decrease in cell viability.

The cellular uptake study indicated that FITC-labeled CNP-L are more efficiently and actively endocytosed by macrophages, which leads to intracellular killing of *M. smegmatis*. The present study concludes that CNP-A and CNP-L group elicit protective immune response against mycobacterial infection in host macrophages. Various CNPs nanoparticles developed in the present study provide a new insight that may help in designing both subunit vaccine as well as anti-tubercular drug delivery system to overcome the problem of drug resistance in Mycobacterial infections.

## Author Contributions

MO was responsible for the origin and design of the experiments. NM designed the experiments. MU and NM conducted the experimental procedures and data analysis. NM and OM wrote the manuscript. All authors approved the final version of the paper.

## Conflict of Interest Statement

The authors declare that the research was conducted in the absence of any commercial or financial relationships that could be construed as a potential conflict of interest. The handling Editor declared a shared affiliation, though no other collaboration with the authors.

## References

[B1] Adams K. N. Takaki K. Connolly L. E. Wiedenhoft H. Winglee K. Humbert O. (2011). Drug tolerance in replicating mycobacteria mediated by a macrophage-induced efflux mechanism. *Cell* 145 39–53. 10.1016/j.cell.2011.02.022 21376383 PMC3117281

[B2] Agnihotri S. A. Mallikarjuna N. N. Aminabhavi T. M. (2004). Recent advances on chitosan-based micro-and nanoparticles in drug delivery. *J. Control. Release* 100 5–28. 10.1016/j.jconrel.2004.08.010 15491807

[B3] Alex S. M. Rekha M. R. Sharma C. P. (2011). Spermine grafted galactosylated chitosan for improved nanoparticle mediated gene delivery. *Int. J. Pharm.* 410 125–137. 10.1016/j.ijpharm.2011.02.067 21396993

[B4] Altaf M. Miller C. H. Bellows D. S. O’Toole R. (2010). Evaluation of the *Mycobacterium smegmatis* and BCG models for the discovery of *Mycobacterium tuberculosis* inhibitors. *Tuberculosis* 90 333–337. 10.1016/j.tube.2010.09.002 20933470

[B5] Amidi M. Mastrobattista E. Jiskoot W. Hennink W. E. (2010). Chitosan-based delivery systems for protein therapeutics and antigens. *Adv. Drug Deliv. Rev.* 62 59–82. 10.1016/j.addr.2009.11.009 19925837

[B6] Anes E. Peyron P. Staali L. Jordao L. Gutierrez M. G. Kress H. (2006). Dynamic life and death interactions between *Mycobacterium smegmatis* and J774 macrophages. *Cell. Microbiol.* 8 939–960. 10.1111/j.1462-5822.2005.00675.x 16681836

[B7] Bonev B. Hooper J. Parisot J. (2008). Principles of assessing bacterial susceptibility to antibiotics using the agar diffusion method. *J. Antimicrob. Chemother.* 61 1295–1301. 10.1093/jac/dkn090 18339637

[B8] Boulos L. Prevost M. Barbeau B. Coallier J. Desjardins R. (1999). LIVE/DEAD BacLight: application of a new rapid staining method for direct enumeration of viable and total bacteria in drinking water. *J. Microbiol. Methods* 37 77–86. 10.1016/S0167-7012(99)00048-2 10395466

[B9] Chassaing B. Rolhion N. de Vallée A. Salim S. Y. Prorok-Hamon M. Neut C. (2011). Crohn disease–associated adherent-invasive *E. coli* bacteria target mouse and human Peyer’s patches via long polar fimbriae. *J. Clin. Invest.* 121 966–975. 10.1172/JCI44632 21339647 PMC3049390

[B10] De Chastellier C. (2009). The many niches and strategies used by pathogenic mycobacteria for survival within host macrophages. *Immunobiology* 214 526–542. 10.1016/j.imbio.2008.12.005 19261352

[B11] Demotz S. Grey H. M. Sette A. (1990). The minimal number of class II MHC-antigen complexes needed for T cell activation. *Science* 249 1028–1030. 10.1126/science.2118680 2118680

[B12] Dong H. Zhu G. Tamada K. Chen L. (1999). B7-H1, a third member of the B7 family, co-stimulates T-cell proliferation and interleukin-10 secretion. *Nat. Med.* 5 1365–1369. 10.1038/70932 10581077

[B13] Drickamer K. (1992). Engineering galactose-binding activity into a C-type mannose-binding protein. *Nature* 360 183–186. 10.1038/360183a0 1279438

[B14] Dube A. Lemmer Y. Hayeshi R. Balogun M. Labuschagne P. Swai H. (2013). State of the art and future directions in nanomedicine for tuberculosis. *Expert Opin. Drug Deliv.* 10 1725–1734. 10.1517/17425247.2014.846905 24102208

[B15] Eruslanov E. Kusmartsev S. (2010). “Identification of ROS using oxidized DCFDA and flow-cytometry,” in *Advanced Protocols in Oxidative Stress II*, ed. Armstrong D. (Totowa, NJ: Humana Press), 57–72. 10.1007/978-1-60761-411-14 20072909

[B16] Espy M. J. Uhl J. R. Sloan L. M. Buckwalter S. P. Jones M. F. Vetter E. A. (2006). Real-time PCR in clinical microbiology: applications for routine laboratory testing. *Clin. Microbiol. Rev.* 19 165–256. 10.1371/journal.pone.0192291 16418529 PMC1360278

[B17] Fisichella M. Dabboue H. Bhattacharyya S. Saboungi M. L. Salvetat J. P. Hevor T. (2009). Mesoporous silica nanoparticles enhance MTT formazan exocytosis in HeLa cells and astrocytes. *Toxicol. Vitro* 23 697–703. 10.1016/j.tiv.2009.02.007 19254755

[B18] Gan Q. Wang T. (2007). Chitosan nanoparticle as protein delivery carrier—systematic examination of fabrication conditions for efficient loading and release. *Colloids Surf. B Biointerfaces* 59 24–34. 10.1016/j.colsurfb.2007.04.009 17555948

[B19] Hall-Stoodley L. Stoodley P. (2005). Biofilm formation and dispersal and the transmission of human pathogens. *Trends Microbiol.* 13 7–10. 10.1016/j.tim.2004.11.004 15639625

[B20] Hashimoto A. Suenaga K. Gloter A. Urita K. Iijima S. (2004). Direct evidence for atomic defects in graphene layers. *Nature* 430 870–873. 10.1038/nature02817 15318216

[B21] He C. Hu Y. Yin L. Tang C. Yin C. (2010). Effects of particle size and surface charge on cellular uptake and biodistribution of polymeric nanoparticles. *Biomaterials* 31 3657–3666. 10.1016/j.biomaterials.2010.01.065 20138662

[B22] Je J. Y. Kim S. K. (2006). Chitosan derivatives killed bacteria by disrupting the outer and inner membrane. *J. Agric. Food Chem.* 54 6629–6633. 10.1016/j.ijfoodmicro.2004.01.022 16939319

[B23] Ko J. A. Park H. J. Hwang S. J. Park J. B. Lee J. S. (2002). Preparation and characterization of chitosan microparticles intended for controlled drug delivery. *Int. J. Pharm.* 249 165–174. 10.1016/S0378-5173(02)00487-8 12433445

[B24] Kong J. Yu S. (2007). Fourier transform infrared spectroscopic analysis of protein secondary structures. *Acta Biochim. Biophys. Sin.* 39 549–559. 10.1111/j.1745-7270.2007.00320.x 17687489

[B25] Koppolu B. Zaharoff D. A. (2013). The effect of antigen encapsulation in chitosan particles on uptake, activation and presentation by antigen presenting cells. *Biomaterials* 34 2359–2369. 10.1016/j.biomaterials.2012.11.066 23274070 PMC3552013

[B26] Kuo J. H. Jan M. S. Chang C. H. Chiu H. W. Li C. T. (2005). Cytotoxicity characterization of catanionic vesicles in RAW264.7 murine macrophage-like cells. *Colloids Surf. B Biointerfaces* 41 189–196. 10.1016/j.colsurfb.2004.12.008 15737546

[B27] Levitte S. Adams K. N. Berg R. D. Cosma C. L. Urdahl K. B. Ramakrishnan L. (2016). Mycobacterial acid tolerance enables phagolysosomal survival and establishment of tuberculous infection in vivo. *Cell host microbe* 20 250–258. 10.1016/j.chom.2016.07.007 27512905 PMC4985559

[B28] Li X. Kong X. Shi S. Zheng X. Guo G. Wei Y. (2008). Preparation of alginate coated chitosan microparticles for vaccine delivery. *BMC Biotechnol.* 8:89. 10.1186/1472-6750-8-89 19019229 PMC2603011

[B29] Lu C. W. Hung Y. Hsiao J. K. Yao M. Chung T. H. Lin Y. S. (2007). Bifunctional magnetic silica nanoparticles for highly efficient human stem cell labeling. *Nano Lett.* 7 149–154. 10.1021/nl0624263 17212455

[B30] McGreal E. P. Miller J. L. Gordon S. (2005). Ligand recognition by antigen-presenting cell C-type lectin receptors. *Curr. Opin. Immunol.* 17 18–24. 10.1016/j.coi.2004.12.001 15653305 PMC7126011

[B31] Meerak J. Wanichwecharungruang S. P. Palaga T. (2013). Enhancement of immune response to a DNA vaccine against *Mycobacterium tuberculosis* Ag85B by incorporation of an autophagy inducing system. *Vaccine* 31 784–790. 10.1016/j.vaccine.2012.11.075 23228812

[B32] Moghimi S. M. Hunter A. C. Murray J. C. (2005). Nanomedicine: current status and future prospects. *FASEB J.* 19 311–330. 10.5603/GP.a2017.0018 15746175

[B33] Nikaido H. (2003). Molecular basis of bacterial outer membrane permeability revisited. *Microbiol. Mol. Biol. Rev.* 67 593–656. 10.1128/MMBR.67.4.593-656.2003 14665678 PMC309051

[B34] No H. K. Park N. Y. Lee S. H. Meyers S. P. (2002). Antibacterial activity of chitosans and chitosan oligomers with different molecular weights. *Int. J. Food Microbiol.* 74 65–72. 10.1016/S0168-1605(01)00717-6 11929171

[B35] Normington K. Kohno K. Kozutsumi Y. Gething M. J. Sambrook J. (1989). *S. cerevisiae* encodes an essential protein homologous in sequence and function to mammalian BiP. *Cell* 57 1223–1236. 10.1016/0092-8674(89)90059-7 2661019

[B36] Prego C. Paolicelli P. Díaz B. Vicente S. Sánchez A. González-Fernández Á (2010). Chitosan-based nanoparticles for improving immunization against hepatitis B infection. *Vaccine* 28 2607–2614. 10.1016/j.vaccine.2010.01.011 20096389

[B37] Raafat D. Von Bargen K. Haas A. Sahl H. G. (2008). Insights into the mode of action of chitosan as an antibacterial compound. *Appl. Environ. Microbiol.* 74 3764–3773. 10.1128/AEM.00453-08 18456858 PMC2446574

[B38] Rabea E. I. Badawy M. E. Stevens C. V. Smagghe G. Steurbaut W. (2003). Chitosan as antimicrobial agent: applications and mode of action. *Biomacromolecules* 4 1457–1465. 10.1021/bm034130m 14606868

[B39] Rinaudo M. Milas M. Le Dung P. (1993). Characterization of chitosan. Influence of ionic strength and degree of acetylation on chain expansion. *Int. J. Biol. Macromol.* 15 281–285. 10.1016/0141-8130(93)90027-J 8251442

[B40] Sahariah P. Masson M. (2017). Antimicrobial chitosan and chitosan derivatives: a review of the structure–activity relationship. *Biomacromolecules* 18 3846–3868. 10.1021/acs.biomac.7b01058 28933147

[B41] Stepanoviæ S. Vukoviæ D. Dakiæ I. Saviæ B. Švabiæ-Vlahoviæ M. (2000). A modified microtiter-plate test for quantification of staphylococcal biofilm formation. *J. Microbiol. Methods* 40 175–179. 10.1016/S0167-7012(00)00122-6 10699673

[B42] Van Soolingen D. (2001). Molecular epidemiology of tuberculosis and other mycobacterial infections: main methodologies and achievements. *J. Intern. Med.* 249 1–26. 10.1046/j.1365-2796.2001.00772.x 11168781

[B43] Wakamoto Y. Dhar N. Chait R. Schneider K. Signorino-Gelo F. Leibler S. (2013). Dynamic persistence of antibiotic-stressed mycobacteria. *Science* 339 91–95. 10.1126/science.1229858 23288538

[B44] Wang T. Bai J. Jiang X. Nienhaus G. U. (2012). Cellular uptake of nanoparticles by membrane penetration: a study combining confocal microscopy with FTIR spectroelectrochemistry. *ACS nano* 6 1251–1259. 10.1021/nn203892h 22250809

[B45] Weissleder R. Kelly K. Sun E. Y. Shtatland T. Josephson L. (2005). Cell-specific targeting of nanoparticles by multivalent attachment of small molecules. *Nat. Biotechnol.* 23 1418–1423. 10.1038/nbt.1159 16244656

[B46] Wilking J. N. Zaburdaev V. De Volder M. Losick R. Brenner M. P. Weitz D. A. (2013). Liquid transport facilitated by channels in *Bacillus subtilis* biofilms. *Proc. Natl. Acad. Sci. U.S.A.* 110 848–852. 10.1073/pnas.1216376110 23271809 PMC3549102

[B47] Wu W. Wieckowski S. Pastorin G. Benincasa M. Klumpp C. Briand J. P. (2005). Targeted delivery of amphotericin B to cells by using functionalized carbon nanotubes. *Angew. Chem. Int. Ed. Engl.* 44 6358–6362. 10.1002/ange.200501613 16138384

[B48] Yang S. C. Hsueh P. R. Lai H. C. Teng L. J. Huang L. M. Chen J. M. (2003). High prevalence of antimicrobial resistance in rapidly growing mycobacteria in Taiwan. *Antimicrob. Agents Chemother.* 47 1958–1962. 10.3201/eid.1502.080837 12760874 PMC155839

[B49] Zambrano M. M. Kolter R. (2005). Mycobacterial biofilms: a greasy way to hold it together. *Cell* 123 762–764. 10.1016/j.cell.2005.11.011 16325571

