# Corrigendum: Selective Targeting of 4SO_4_-*N*-Acetyl-Galactosamine Functionalized *Mycobacterium tuberculosis* Protein Loaded Chitosan Nanoparticle to Macrophages: Correlation With Activation of Immune System

**DOI:** 10.3389/fmicb.2020.621067

**Published:** 2021-01-20

**Authors:** Nida Mubin, Mohd. Saad Umar, Swaleha Zubair, Mohammad Owais

**Affiliations:** ^1^Interdisciplinary Biotechnology Unit, Aligarh Muslim University, Aligarh, India; ^2^Department of Computer Science, Aligarh Muslim University, Aligarh, India

**Keywords:** Acr-1 (Rv2031c), *M. smegmatis*, RAW264.7, 4-SO_4_-GalNAc, CNPs

In the original article, there was a mistake in Figure 5 as published. The figure panels were arranged incorrectly. The corrected Figure 5 appears below.

**Figure 5:**
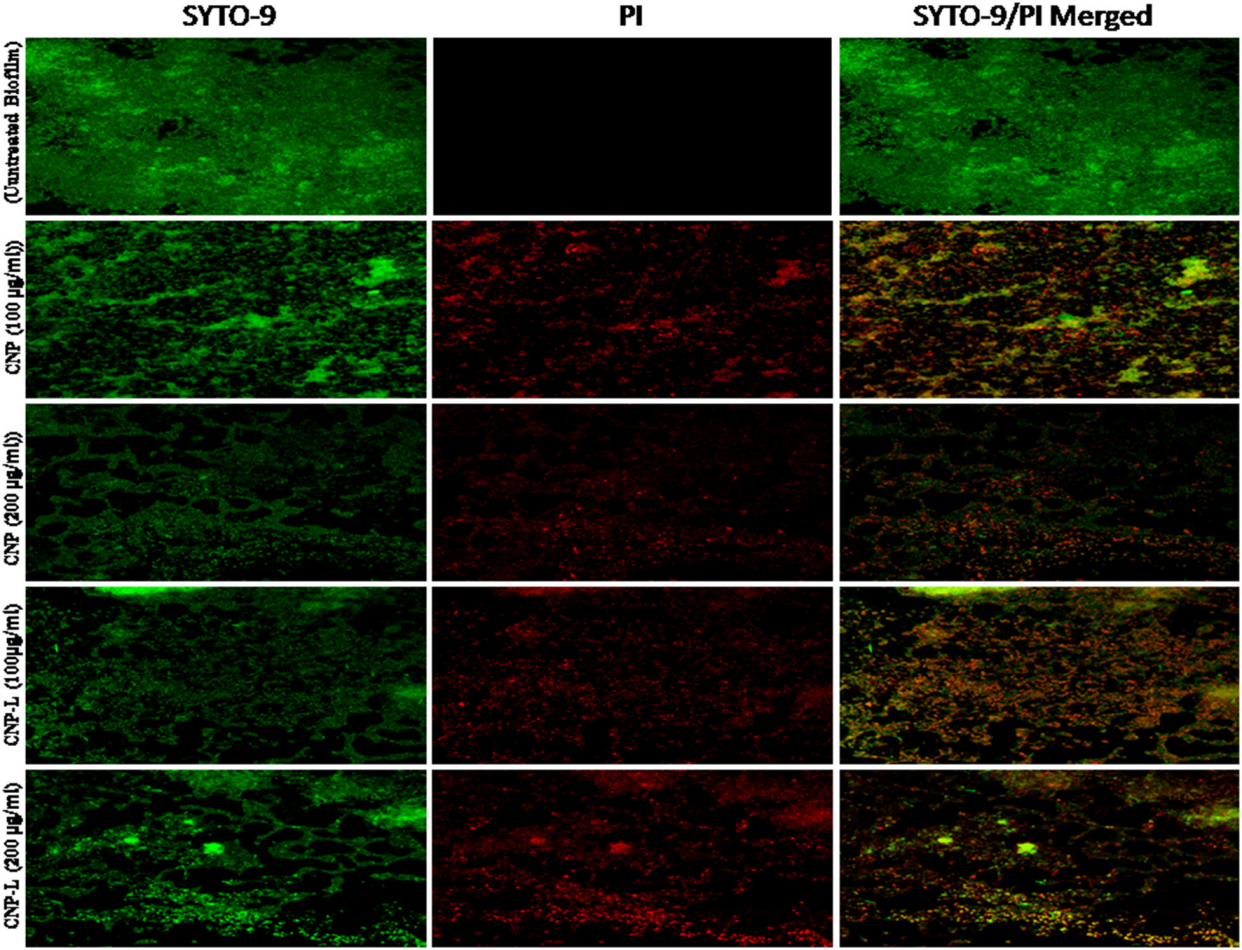
Confocal microscope image showing *M. smegmatis* biofilm inhibition by CNPs as visualized in 63X oil immersion magnification: anti-biofilm activity of CNPs was assessed by incubating *M. smegmatis* with increasing concentrations of CNPs for 36 h in a six-well plate. The treated biofilm was stained with SYTO-9/PI. The addition of increasing concentration (100–200 μg/ml) of CNPs inhibited *M. smegmatis* biofilm formation. Red-dye showing PI-stain corresponds to killing activity, Green dye showing viable bacteria in pre formed biofilm. Yellow color corresponds to co-localization of merged green and red dye at same place.

The authors apologize for this error and state that this does not change the scientific conclusions of the article in any way. The original article has been updated.

