# Supplementary figures and images for: Selective Targeting of 4SO4-N-Acetyl-Galactosamine Functionalized Mycobacterium tuberculosis Protein Loaded Chitosan Nanoparticle to Macrophages: Correlation With Activation of Immune System
Source: Front Microbiol. 2018 Nov 20;9:2469. doi: 10.3389/fmicb.2018.02469 (PMC6255963; doi:10.3389/fmicb.2018.02469)

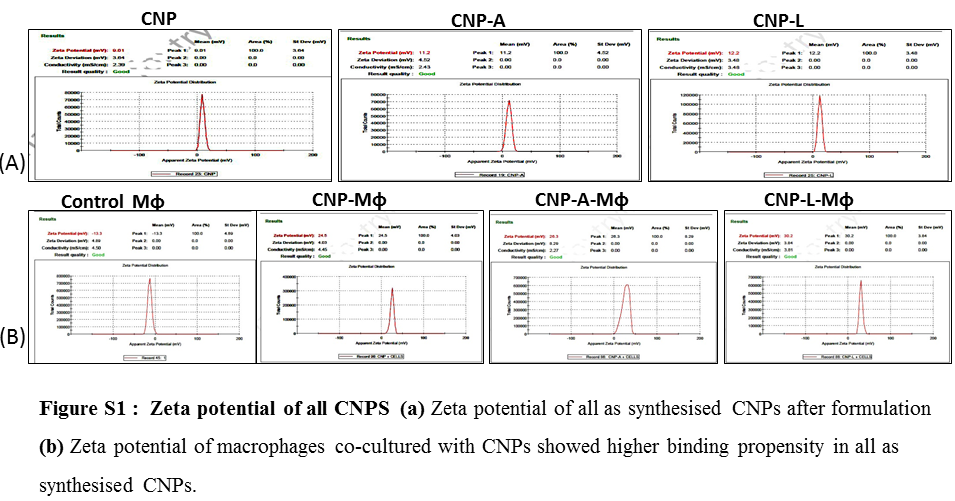

Supplement: Supplementary file 1 [file Image_1.tif]

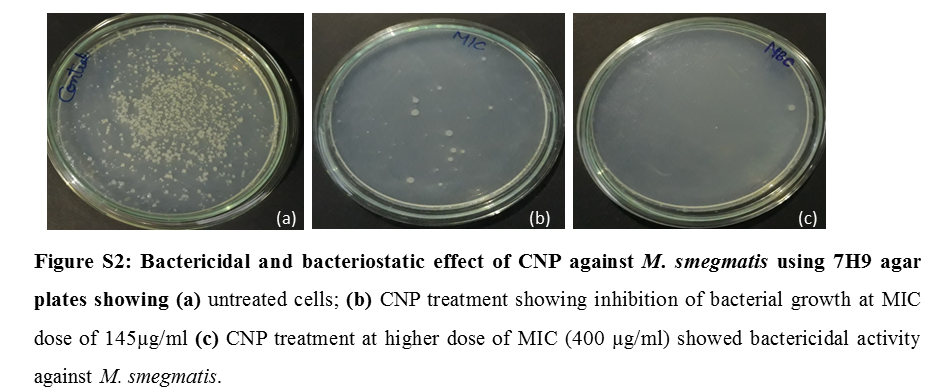

Supplement: Supplementary file 2 [file Image_2.tif]
